# Cyclopædia of Practical Medicine

**Published:** 1845-06-01

**Authors:** 


CYCLOPÆDIA OF PRACTICAL MEDICINE.—This comprehensive work, edited by Drs. Forbes, Tweedie and Conolly, assisted by a host of the most eminent of the British writers, each contributing articles on subjects with which his own labors have become especially identified, has lately been republished in this country by Lea and Blanchard of Philadelphia, under the supervision of Prof. Dunglison.

The Cyclopædia must be regarded as the most complete work of practical medicine extant; or at least in our language. The amount of information on every topic which it embraces, is posted up to the present time; and so far as we are able to judge, it is generally more free from national exclusiveness and prejudices, than is usually the case with British publications. We are not prepared to institute any critical comparision between this and the corresponding French work—the *Dictionnaire de Medicine.* The latter which is much more extensive and elaborate, and is still incomplete, has been so long in course of publication, that the earlier portions, are already far in the rear of the present state of science. This must be a serious objection since the difficulty can be but partially remedied by new editions.

Prof. Dunglison, the American editor of the Cyclopædia, has added considerable matter, bringing it still more closely to the present time.

The getting up of the American edition is very creditable to the Publishers. It will compare very favorably with the English edition. In some respects, it is much to be preferred. During the original publication, many of the articles not being in readiness to be printed in proper alphabetical order, it became necessary to include them together in a single volume as a supplement to the work. This difficulty is obviated in the American edition. On the whole, we advise those who desire a compendious collection of the latest and most important information in the various departments of practical medicine—including midwifery, materia medica, medical jurisprudence, &c., to possess themselves of this work.

Among such a mass of valuable matter, it would be a difficult as well as invidious task to indicate particular excellencies. We would, however, refer the reader to the "History of Medicine from the termination of the eighteenth century to the present time," for an admirable digest of the characteristic traits of Modern Medicine, from the pen of one of the most philosophical and polished writers of the present day, Dr. Alison.

